# Drug Susceptibility Testing and Synergistic Antibacterial Activity of Curcumin with Antibiotics against Enterotoxigenic *Escherichia coli*

**DOI:** 10.3390/antibiotics8020043

**Published:** 2019-04-18

**Authors:** Rangel-Castañeda Itzia Azucena, Cruz-Lozano José Roberto, Zermeño-Ruiz Martin, Cortes-Zarate Rafael, Hernández-Hernández Leonardo, Tapia-Pastrana Gabriela, Castillo-Romero Araceli

**Affiliations:** 1 Departamento de Fisiología, Centro Universitario de Ciencias de la Salud, Universidad de Guadalajara, Calle Sierra Mojada 950, Independencia Oriente, Guadalajara 44340, Jalisco, Mexico; itzia.rangel2591@gmail.com (R.-C.I.A.); leohhdez@hotmail.com (H.-H.L.); 2 Departamento de Química, Centro Universitario de Ciencias Exactas e Ingenierías, Universidad de Guadalajara, Blvd. Marcelino García Barragán 1421, Guadalajara 44430, Jalisco, Mexico; robertocrz@hotmail.com; 3 Departamento de Microbiología y Patología, Centro Universitario de Ciencias de la Salud, Universidad de Guadalajara, Calle Sierra Mojada 950, Independencia Oriente, Guadalajara 44340, Jalisco, Mexico; martinzermeno@hotmail.com (Z.-R.M.); rcortesz@hotmail.com (C.-Z.R.); 4 Hospital Regional de Alta Especialidad de Oaxaca, Calle Aldama S/N, 71256 San Bartolo Coyotepec, Oax, Mexico; gabrielatapiapastrana@gmail.com

**Keywords:** curcumin, antibacterial, antibiotics, synergism, *Escherichia coli*

## Abstract

**Aim:** This study investigated the susceptibility of Enterotoxigenic *Escherichia coli* to curcumin, as well as its synergistic effect with 12 antimicrobial drugs. **Methods and Results:** Our study shows that curcumin did not affect bacterial growth. The antimicrobial susceptibility of curcumin and antibiotic synergy were identified using disc diffusion on Mueller-Hinton agar. The strain of Enterotoxigenic *Escherichia coli* used was resistant to Ampicillin, Amoxicillin/Clavulanic acid, Ampicillin/Sulbactam, Ciprofloxacin, and Cefazolin. There was synergy between curcumin and the majority of antibiotics tested. Maximum synergy was observed with combinations of 330 µg/mL curcumin and Ceftazidime, followed by Cefotaxime, Amoxicillin/Clavulanic acid, Ampicillin, Aztreonam, Trimethoprim, Ciprofloxacin, Ceftriaxone, Cefazolin, Tetracycline, and Imipenem. **Conclusion:** Our findings indicated that curcumin might be useful as a combinatorial strategy to combat the antibiotic resistance of Enterotoxigenic *Escherichia coli*.

## 1. Introduction

Acute diarrheal disease (ADD) is an important health problem globally, particularly in the youngest children in resource-limited (developing) countries [1]. The etiological agents most frequently involved vary according to the socioeconomic status and health conditions of the region. In Mexico, diarrheagenic *Escherichia coli* strains have been reported as the most prevalent etiologic agents for this disease [2,3]. Enterotoxigenic *E. coli* (ETEC) is one of the main causes of illness and mortality in children under five years of age, is the leading cause of traveler’s diarrhea, and is endemic in essentially all developing countries [4,5]. The vast majority of cases with severe or persistent diarrhea are treated empirically without bacteriological investigation [6]. *E coli* enteric infections require fluid replacement with solutions containing appropriate electrolytes to prevent dehydration. However, antimicrobial therapy should be used in severe cases of diarrheal disease and may be used to prevent traveler’s diarrhea. Antimicrobials known to be useful include doxycycline, trimethoprim/sulfamethoxazole, fluoroquinolones, and rifaximin [7]. Unfortunately, the antibiotic resistance crisis is becoming dire, and *E. coli* has already shown resistance to fluoroquinolones, carbapenems, and even third-generation cephalosporin’s ?the best antibiotics available to treat multi-resistant-bacteria [8,9,10,11]. *E. coli* is included in the group of bacteria of critical priority for the development of new antibiotics [12]. In the search for new alternative therapies for treating ADD, natural products are being evaluated. Among those, curcumin (CUR), the main biologically active curcuminoid of *Curcuma longa* L., possesses a wide spectrum of biological activities, such as antioxidant, anti-inflammatory, anti-tumor, anti-proliferative, and anti-protozoal properties [13,14,15,16]. CUR alone or combined with some nanomaterials has demonstrated an antibacterial property against both Gram-positive and Gram-negative bacteria, such as *E. coli* [17,18,19,20,21,22]. However, its effect on Enterotoxigenic *E. coli* is unknown. On the other hand, CUR has received worldwide attention as a combinatorial therapy; its antibacterial action is synergistic with several antibiotics [23,24]. In this study, we analyzed the effect of CUR in a strain of ETEC and its antibacterial synergy with 12 potentially useful agents, in order to contribute to the establishment of a combinatorial strategy that may be more effective for treating the disease.

## 2. Results

### 2.1. Curcumin Did not Affect the Growth of Enterotoxigenic E. coli

**Figure 1:**
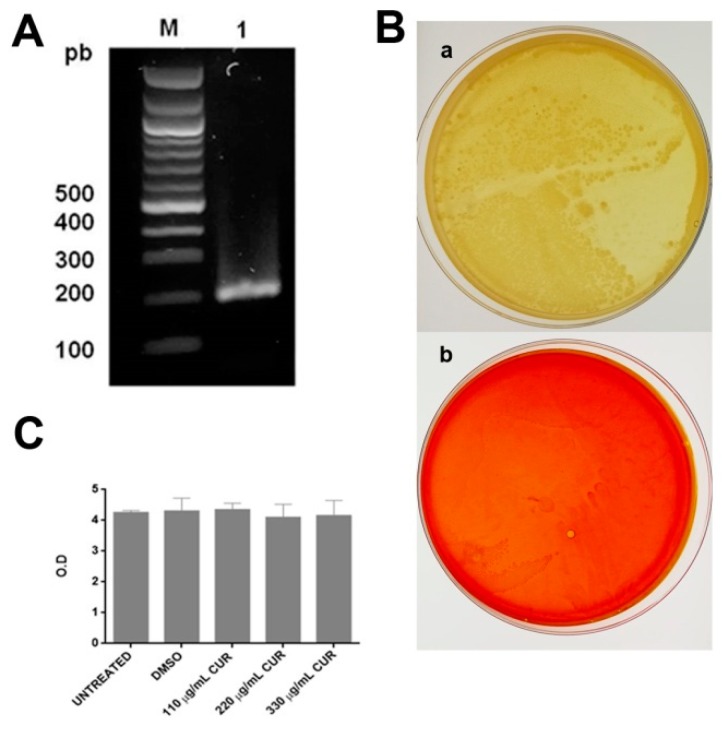
(**A**) PCR amplification of the gene for the heat-labile toxin, a virulence factor associated with the ETEC: lane 1 (218 bp), M (Molecular weight marker). (**B**) Representative images of the effect of CUR on the growth of ETEC, (**a**) DMSO, and (**b**) CUR 330 µg/mL. (**C**) The growth curve of ETEC (optical density 600 nm) in media; DMSO; and 110, 220, or 330 μg/mL of CUR. Data correspond to mean values ± SD of three independent experiments.

The classification of the strain as an ETEC was validated as the strain possessed the heat-labile toxin (LT). Figure 1A shows the electrophoresis pattern of the PCR product for the LT gene obtained. This product was 218 pb, which was in agreement with the size reported earlier [25]. For the antimicrobial susceptibility tests to CUR, agar dilution and broth microdilution methods were used. The results showed that treatment of ETEC with 110, 220, or 330 µg/mL of CUR did not inhibit bacterial growth; on agar plates, many colony-forming units were observed, and the individual colonies were blended together, making them impossible to count (Figure 1B). As shown in Figure 1C, the broth microdilution method confirmed that CUR had no activity against ETEC, as no significant differences in growth rates (OD600) were observed when grown in DMSO or CUR.

### 2.2. Susceptibility of Enterotoxigenic E. coli

The antibiotic susceptibility of the ETEC strain was assessed by disk diffusion using 12 different antimicrobial agents (Table 1). Our data evidenced that ETEC was resistant to Ampicillin, Ampicillin/Sulbactam, Amoxicillin/Clavulanic acid, and Cefazolin, but was sensitive to all other antibiotics, with the exception of Ciprofloxacin, where the strain showed intermediate resistance (Table 2).

### 2.3. Synergistic Effect of Curcumin

**Figure 2:**
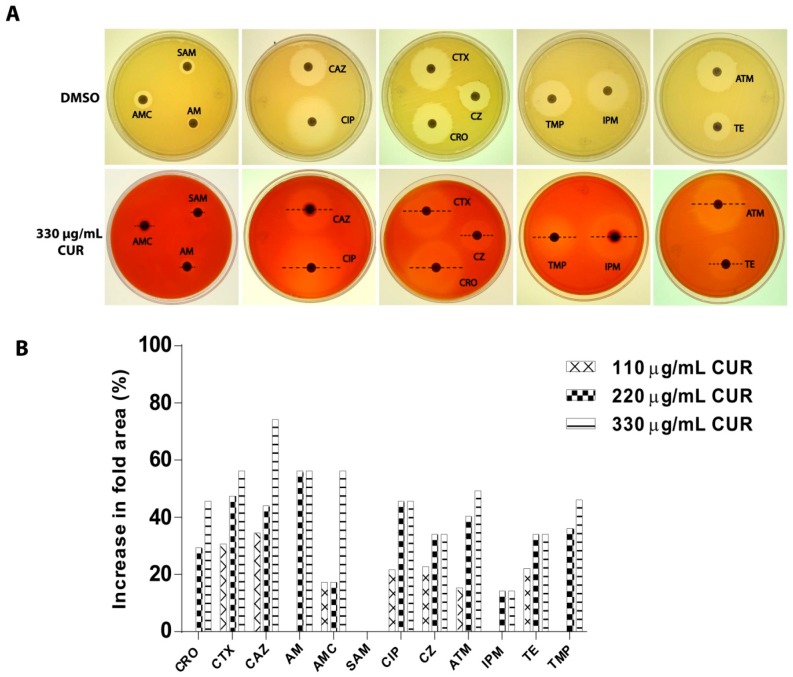
Synergistic effect of curcumin. (**A**) Representative images of susceptibility at different antibiotics against a strain of Enterotoxigenic *E. coli* (in the absence or presence of curcumin). (**B**) The percent of increases in inhibition area for different antibiotics was calculated as (B^2^−A^2^)/A^2^ × 100, where A is the inhibition zone in the absence of curcumin and B is the inhibition zone in the presence of curcumin. See Table 3 for A and B values.

The possible synergistic effect of CUR was evaluated. CUR at 110, 220, or 330 µg/mL enhanced the antibacterial activity of the majority of the tested antibiotics (Table 3). These effects were most dramatic with 330 µg/mL of CUR, and the highest fold increases were observed for Ceftazidime (74.2%), followed by Amoxicillin/Clavulanic acid (56.2%), Cefotaxime (56.2%), and Ampicillin (56.2%) (Figure 2).

## 3. Discussion

The clinical threat of infections with antibiotic-resistant bacteria is increasing [26]. *E. coli* is found in humans and other mammals as a commensal microorganism. However, some *E. coli* strains are responsible for diverse intestinal and extraintestinal diseases. The Enterotoxigenic *E. coli* ETEC is one of the main causes of illness and mortality in children under five years of age, and is the leading cause of traveler’s diarrhea; it is endemic in essentially all developing countries [3,4]. According to the global disease burden estimates and the Maternal and Child Epidemiology Estimation group (MCEE), although mortality rates from diarrheal diseases have decreased since 1900, ETEC ranks among the top five causes of diarrheal worldwide [27,28]. This pathogen in increasingly resistant to antibiotics, and its treatment is generally ineffective [4,29,30]. To solve this problem, there is an urgent need to develop or discover new antibacterials. CUR has been known for a long time for its antimicrobial properties [19,20,31]. Previous reports described that CUR has high antibacterial activity against a commensal *E. coli*, strain ATCC 25922, in relation to other bacteria, with a minimum inhibitory concentration (MIC) for CUR of 163 µg/mL [19]. In the present study, CUR, even up to 330 µg/mL, was not active against the strain of ETEC used. This result is consistent with other reports which showed that the ethanol extract of the rhizome of *Curcuma longa*, as well as three curcuminoids isolated from the same plant, do not possess antibacterial activity against *E. coli*. This has been attributed to the composition of the outer membrane of Gram-negative strains [21,31]. 

On the other hand, several studies have demonstrated that a combination of therapies of antibiotics and other substances might improve the therapeutic effect and reduce drug resistance [32]. CUR has been shown to increase the activity of several antibiotics against different bacteria [23,33]. The current study demonstrated that CUR in combination with several antibiotics was synergistic. By the disk diffusion method assay, CUR showed enhancement of the inhibition zone with almost all the drug combinations, except with Ampicillin/Sulbactam. This finding is further supported by other studies on Gram-negative and Gram-positive bacteria, which reported the synergistic antibacterial activity of CUR with antibiotics [24,34]. We propose that CUR could be of relevance in combination therapy, and may be useful as a treatment option for drug-resistant bacteria.

## 4. Materials and Methods

### 4.1. Bacterial Strain

The enterotoxigenic *E. coli* (H10407 prototypical ETEC 078:H11) used in the current study was kindly provided by Dr. Fernando Navarro García, Center for Research and Advanced Studies of the National Polytechnic Institute (CINVESTAV).

### 4.2. Maintenance and Preservation of Microorganisms

The enterotoxigenic E. coli strain H10407 was grown on nutrient agar plates (Becton Dickinson, Maryland, USA) at 37 °C for 18–20 h. The cultures were stored at 4 °C and were re-cultured every seven days. 

### 4.3. Identification of the Presence of the Gene of the Heat-labile Toxin in Enterotoxigenic E. coli by PCR

#### 4.3.1. Extraction of Genomic DNA

Cells were harvested by centrifugation at 5000 rpm for 3 min. DNA extraction was performed using the DNeasy^®^ Blood & Tissue kit (QIAGEN, Hilden, Germany), following the manufacturer’s instructions. The DNA was stored at −20 °C. Purity and concentration were determined by electrophoresis in 1% agarose gel (Ultra-Pure™ Agarose, Invitrogen, Carlsbad, California USA) and by spectrophotometry, respectively. The samples were stained with GelRed™ (Biotium, Landing Pkwy, California USA) and the bands were visualized in a trans-illuminator (UVP Benchtop 2UV, Fisher Scientific, Waltham, Massachusetts, USA).

#### 4.3.2. Expression of LT Gene

The heat-labile toxin (LT) is one of the major virulence factors of enterotoxigenic *Escherichia coli* [25]. Expression of LT (GenBank Accession MF374714.1) was validated in the ETEC strain (Figure 1A). The gene was amplified from genomic DNA by PCR using the following primers: lt sense 5’-GCA CAC GGA GCT CCT CAG TC-3’ and lt antisense 5’-TCC TTC ATC CTT TCA ATG GCT TT-3’. The PCR conditions were: hot starts 94 °C for 5 min, 35 cycles at 94 °C for 90 s, 58 °C for 90 s, and 72 °C for 90 s. The resulting amplicons were visualized by electrophoresis in 1% agarose gel.

### 4.4. Antibacterial Effect of Curcumin

#### 4.4.1. Preparation of Curcumin Stocks

The CUR was obtained from Sigma Chemicals Co. (St Louis, Missouri, USA). The stock of CUR was prepared to a concentration of 40 mg/mL, dissolved in dimethyl sulfoxide (DMSO, Sigma), and diluted with Mueller-broth media (Becton Dickinson, Maryland, USA) to a concentration of 110, 220, or 330 µg/mL.

#### 4.4.2. Killing Assays

To determine the possible effect of CUR on the growth of ETEC, the agar plate method and microdilution technique were used [35,36]. Briefly, 100 µL of bacterial suspension with an OD600 of 0.4 (3.2 × 10^8^ CFU/mL) was streaked on Mueller-Hinton agar (MHA) (MCDLAB, Tlalnepantla, Mexico state), containing 110, 220, or 330 µg/mL of CUR. The plates were incubated for 24 h at 37 °C. After incubation, the bacterial cell growth on the solid surface was observed directly. For the microdilution assay, 100 µL of Mueller-broth media (MBM) supplemented with 110, 220, or 330 µg/mL of CUR was dispensed in each well. The wells were inoculated with 1 µL of bacterial suspension with an OD600 of 0.4 (3.2 × 10^8^ CFU/mL). Cultures without CUR or with DMSO 1.27% were used as negative controls. The plates were incubated for 24 h at 37 °C. After incubation, bacterial growth (OD600) was measured on a microplate reader (BioTek Synergy HT, Winooski, Vermont, USA). All experiments were performed in triplicate.

### 4.5. Determination of Bacterial Susceptibility to Antibiotics

To determine the antimicrobial susceptibility of ETEC, the agar diffusion method was used. Briefly, the bacterial suspension was prepared by suspending three to four well-separated overnight colonies into 3 mL of MBM. Cells were incubated at 37 °C and 250 rpm, and the growth was monitored until an OD600 of 0.4 (3.2 × 10^8^ CFU/mL) was reached. The suspension was used to inoculate by swabbing plates of MHA. The plates were allowed to dry for 5 min, and twelve commercially-prepared paper antibiotic disks (Table 1) were then placed on the inoculated agar surfaces. Plates were incubated at 37 °C for 20 h. The zones of growth inhibition around each antibiotic disk were measured by a Vernier caliper. The diameter of each zone was interpreted using the criteria published by the Clinical and Laboratory Standards Institute (CLSI manual for antimicrobial susceptibility, 2017).

### 4.6. Synergistic Effect of Curcumin on a Strain of Enterotoxigenic E. coli

To determinate whether CUR had a synergistic effect with twelve potentially anti-ETEC agents, the agar diffusion method was used. Briefly, MHA agar plates incorporating 110, 220, or 330 µg/mL of CUR were inoculated with bacterial suspensions described above using a sterile swab, following CLSI recommendations. The plates were incubated under the same condition as for disc diffusion. The sensi-discs were placed and incubated at 37 °C for 20 h. Plates without CUR and DMSO 1.27% were used as negative controls. The zones of growth inhibition around each antibiotic disk were measured by a Vernier caliper.

### 4.7. Statistical Analysis

All data were presented as mean values with standard deviations and analyzed using two-way ANOVA followed by Dunnett’s multiple comparisons test (GraphPad Prism version 6.01 for Windows, GraphPad Software, La Jolla California USA), and *p* values of ≤0.05 were considered significantly different.

## 5. Conclusions

Our results are the first study to investigate the effect of CUR on enterotoxigenic *Escherichia coli* ETEC, and demonstrate that CUR did not affect the growth of ETEC. CUR improved the activity of the 12 commercial antibiotics tested, exhibiting good synergism against ETEC. Further studies are needed to establish CUR as complementary therapy against diarrheagenic *E. coli* strains or multi-drug-resistant strains and to identify the mechanisms of the synergistic effect.

## Figures and Tables

**Table 1:** Antibiotics used in the susceptibility tests and the break-points for resistance and susceptibility.

Antibiotic	Concentration (μg)	Inhibition Halo (mm)		
		Sensitive (S)	Intermediate (I)	Resistant (R)
Ceftriaxone (CRO)	30	≥23	20–22	≤ 19
Cefotaxime (CTX)	30	≥26	23–55	≤ 22
Ceftazidime (CAZ)	30	≥21	18–20	≤ 17
Ampicillin (AM)	10	≥17	14–16	≤ 13
Amoxicillin/Clavulanic acid (AMC)	20/10	≥18	14–17	≤ 13
Ampicillin/Sulbactam (SAM)	10/10	≥15	12–14	≤ 11
Ciprofloxacin (CIP)	5	≥31	21–30	≤ 20
Cefazolin (CZ)	30	≥23	20- 22	≤ 19
Aztreonam (ATM)	30	≥21	18–20	≤ 17
Imipinem (IPM)	10	≥23	20–22	≤ 19
Tetracycline (TE)	30	≥15	12–14	≤ 11
Trimethoprim (TMP)	5	≥16	11–15	≤ 10

**Table 2:** Zone of inhibition (mm) and susceptibility of different antibiotics against Enterotoxigenic *Escherichia coli*.

Antibiotics	Only Antibiotic
Ceftriaxone	29 ± 1.0 (S)
Cefotaxime	28 ± 1.2(S)
Ceftazidime	25 ± 1.2 (S)
Ampicillin	8 ± 1.0 (R)
Amoxicillin/ Clavulanic acid	12 ± 1.0 (R)
Ampicillin/Sulbactam	11 ± 1.0 (R)
Ciprofloxacin	29 ± 1.0 (I)
Cefazolin	19 ± 1.2 (R)
Aztreonam	27 ± 1.0 (S)
Imipinem	29 ± 1.2 (S)
Tetracycline	19 ± 1.0 (S)
Trimethomprim	24 ± 0.6 (S)

S = sensitive, I = intermediate and R = resistant.

**Table 3:** Zone of inhibition (mm) and susceptibility of different antibiotics against a strain of Enterotoxigenic *Escherichia coli* in the absence and presence of curcumin.

Antibiotics	Antibiotic + DMSO ^A^	Antibiotic + 110 µg/mL CUR ^B^	Antibiotic + 220 µg/mL CUR ^B^	Antibiotic + 330 µg/mL CUR ^B^
Ceftriaxone	29 ± 1.0 (S)	29 ± 1.0 (S)	33 ± 1.2 (S)	35 ± 1.0 (S)
Cefotaxime	28 ± 1.0 (S)	32 ± 0.6 (S)	34 ± 1.0 (S)	35 ± 1.0 (S)
Ceftazidime	25 ± 1.2 (S)	29 ± 1.0 (S)	30 ± 1.0 (S)	33 ± 0.6 (S)
Ampicillin	8 ± 1.0 (R)	8 ± 0.0 (R)	10 ± 0.6(R)	10 ± 0.0 (R)
Amoxicillin/Clavulanic acid	12 ± 1.2 (R)	13 ± 1.0 (R)	13 ± 1.0 (R)	15 ± 1.0 (I)
Ampicillin/Sulbactam	11 ± 0.0 (R)	11 ± 0.6 (R)	11 ± 1.0 (R)	11 ± 1.0 (R)
Ciprofloxacin	29 ± 1.0 (I)	32 ± 1.0 (S)	35 ± 1.0 (S)	35 ± 1.2 (S)
Cefazolin	19 ± 1.2 (R)	21 ± 1.0 (I)	22 ± 1.0 (I)	22 ± 0.6 (I)
Aztreonam	27 ± 0.0 (S)	29 ± 0.6 (S)	32 ± 0.6 (S)	33 ± 1.0 (S)
Imipinem	29 ± 0.6 (S)	29 ± 1.2 (S)	31 ± 0.6 (S)	31 ± 0.0 (S)
Tetracycline	19 ± 1.0 (S)	21 ± 1.0 (S)	22 ± 0.6 (S)	22 ± 0.6 (S)
Trimethomprim	24 ± 0.6 (S)	24 ± 1.0 (S)	28 ± 1.0 (S)	29 ± 1.0 (S)

^A^ is the inhibition zone in the absence of curcumin. ^B^ is the inhibition zone in the presence of curcumin. S = sensitive, I = intermediate, and R = resistant.

## References

[1] Lanata C.F., Fischer-Walker C.L., Olascoaga A.C., Torres C.X., Aryee M.J., Black R.E., Child Health Epidemiology Reference Group of the World Health Organization, UNICEF (2013). Global causes of diarrheal disease mortality in children <5 years of age: A systematic review. PLoS ONE.

[2] Canizalez-Roman A., Flores-Villaseñor H.M., Gonzalez-Nuñez E., Velazquez-Roman J., Vidal J.E., Muro-Amador S., Alapizco-Castro G., Díaz-Quiñonez J.A., León-Sicairos N. (2016). Surveillance of diarrheagenic *Escherichia coli* strains isolated from diarrhea cases from children, adults and elderly at northwest of Mexico. Front Microbiol..

[3] Navarro A., Estrada-Garcia T., Tores G.A. (2010). Epidemiology of diarrheagenic *Escherichia coli* pathotypes in Mexico, past and present. Pathogenic Escherichia coli in Latin America.

[4] Qadri F., Svennerholm A.M., Faruque A.S., Sack R.B. (2005). Enterotoxigenic *Escherichia coli* in developing countries: Epidemiology, microbiology, clinical features, treatment, and prevention. Clin. Microbiol. Rev..

[5] Okoh A.I., Osode A.N. (2008). Enterotoxigenic *Escherichia coli* (ETEC): A recurring decimal in infants’ and travelers’ diarrhea. Rev. Environ. Health.

[6] Pawlowski S.W., Warren C.A., Guerrant R. (2009). Diagnosis and treatment of acute or persistent diarrhea. Gastroenterology.

[7] DuPont H.L. (2005). Travelers’ diarrhea: Antimicrobial therapy and chemoprevention. Nat. Clin. Pract. Gastroenterol. Hepatol..

[8] Tagajdid M.R., Boumhil L., Iken M., Adnaoui M. (2010). Resistance to fluoroquinolones and third generation cephalosporin of *Escherichia coli* isolated from urines. Med. Mal. Infect..

[9] Estrada-Garcia T., Cerna J.F., Paheco-Gil L., Velázquez R.F., Ochoa T.J., Torres J., DuPont H.L. (2005). Drug-resistant diarrheogenic *Escherichia coli*, Mexico. Emerg. Infect. Dis..

[10] Camins B.C., Marschall J., DeVader S.R., Maker D.E., Hoffman M.W., Fraser V.J. (2011). The clinical impact of fluoroquinolone resistance in patients with *E coli* bacteremia. J. Hosp. Med..

[11] Liang W.J., Liu H.Y., Duan G.C., Zhao Y.X., Chen S.Y., Yang H.Y., Xi Y.L. (2018). Emergence and mechanism of carbapenem-resistant *Escherichia coli* in Henan, China, 2014. J. Infect. Public Health.

[12] World Health Organization (2017). Antibiotic-Resistant “Priority Pathogens”.

[13] de Paula Aguiar D., Brunetto Moreira Moscardini M., Rezende Morais E., Graciano de Paula R., Ferreira P.M., Afonso A., Belo S., Tomie Ouchida A., Curti C., Cunha W.R. (2016). Curcumin generates oxidative stress and induces apoptosis in adult *Schistosoma mansoni* worms. PLoS ONE.

[14] Gutierrez-Gutierrez F., Palomo-Ligas L., Hernández-Hernández J.M., Pérez-Rangel A., Aguayo-Ortiz R., Hernández-Campos A., Castillo R., González-Pozos S., Cortés-Zárate R. (2017). Curcumin alters the cytoskeleton and microtubule organization on trophozoites of *Giardia lamblia*. Acta Trop..

[15] Aggarwal B.B., Sundaram C., Malani N., Ichikawa H. (2007). Curcumin: The Indian solid gold. Adv. Exp. Med. Biol..

[16] Zhang Y., Zeng Y. (2019). Curcumin reduces inflammation in knee osteoarthritis rats through blocking TLR4 /MyD88/NF-kappaB signal pathway. Drug Dev. Res..

[17] Teow S.Y., Liew K., Ali S.A., Khoo A.S., Peh S.C. (2016). Antibacterial action of curcumin against *Staphylococcus aureus*: A brief review. J. Trop. Med..

[18] Hu P., Huang P., Chen M.W. (2013). Curcumin reduces *Streptococcus mutans* biofilm formation by inhibiting sortase A activity. Arch. Oral. Biol..

[19] Gunes H., Gulen D., Mutlu R., Gumus A., Tas T., Topkaya A.E. (2016). Antibacterial effects of curcumin: An in vitro minimum inhibitory concentration study. Toxicol. Ind. Health.

[20] De R., Kundu P., Swarnakar S., Ramamurthy T., Chowdhury A., Nair G.B., Mukhopadhyay A.K. (2009). Antimicrobial activity of curcumin against *Helicobacter pylori* isolates from India and during infections in mice. Antimicrob. Agents Chemother..

[21] García-Ariza L., Sierra-Acevedo O.-M.J., Padilla-Sanabria L. (2017). Biological activity of three curcuminoids from *Curcuma longa* L. (turmeric) grown un Quíndio, Colombia. Rev. Cubana Plant Med..

[22] Tyagi P., Singh M., Kumari H., Kumari A., Mukhopadhyay K. (2015). Bactericidal activity of curcumin I is associated with damaging of bacterial membrane. PLoS ONE.

[23] Sasidharan N.K., Sreekala S.R., Jacob J., Nambisan B. (2014). In vitro synergistic effect of curcumin in combination with third generation cephalosporins against bacteria associated with infectious diarrhea. Biomed. Res. Int..

[24] Kali A., Kali A., Bhuvaneshwar D., Charles P.M.V., Srinivasaiah Seetha K. (2016). Antibacterial synergy of curcumin with antibiotics against biofilm producing clinical bacterial isolates. J. Basic Clin. Pharm..

[25] Rajendran P., Ajjampur S.S., Chidambaram D., Chandrabose G., Thangaraj B., Sarkar R., Samuel P., Rajan D.P., Kang G. (2010). Pathotypes of diarrheagenic *Escherichia coli* in children attending a tertiary care hospital in South India. Diagn. Microbiol. Infect. Dis..

[26] Mave V., Chandanwale A., Kagal A., Khadse S., Kadam D., Bharadwaj R., Dohe V., Robinson M.L., Kinikar A., Joshi S. (2017). High burden of antimicrobial resistance and mortality among adults and children with community-onset bacterial infections in India. J. Infect. Dis..

[27] GBD Diarrhoeal Disease Collaborators (2018). Estimates of the global, regional, and national morbidity, mortality, and aetiologies of diarrhoea in 195 countries: A systematic analysis for the Global Burden of Disease Study 2016. Lancet Infect. Dis..

[28] GBD Diarrhoeal Disease Collaborators (2017). Estimates of global, regional, and national morbidity, mortality, and aetiologies of diarrhoeal diseases: A systematic analysis for the Global Burden of Disease Study 2015. Lancet Infect. Dis..

[29] Hong K.S., Kim J.S. (2011). Rifaximin for the treatment of acute infectious diarrhea. Therap. Adv. Gastroenterol..

[30] Chakraborty S., Deokule J.S., Garg P., Bhattacharya S.K., Nandy R.K., Balakrish Nair G., Yamasaki S., Takeda Y., Ramamurthy T. (2001). Concomitant infection of enterotoxigenic *Escherichia coli* in an outbreak of cholera caused by *Vibrio cholerae* O1 and O139 in Ahmedabad, India. J. Clin. Microbiol..

[31] Tajbakhsh S., Deilami M.K., Zandi K., Fouladvand M., Ramedani E., Asayeshm G. (2008). Antibacterial activity of indium curcumin and indium diacetylcurcumin. Afr. J. Biotechnol..

[32] Ejim L., Farha M.A., Falconer S.B., Wildenhain J., Coombes B.K., Tyers M., Brown E.D., Wright G.D. (2011). Combinations of antibiotics and nonantibiotic drugs enhance antimicrobial efficacy. Nat. Chem. Biol..

[33] Roudashti S., Zeighami H., Mirshahabi H., Bahari S., Soltani A., Haghi F. (2017). Synergistic activity of sub-inhibitory concentrations of curcumin with ceftazidime and ciprofloxacin against *Pseudomonas aeruginosa* quorum sensing related genes and virulence traits. World J. Microbiol. Biotechnol..

[34] Teow S.Y., Ali S.A. (2015). Synergistic antibacterial activity of Curcumin with antibiotics against *Staphylococcus aureus*. Pak. J. Pharm. Sci..

[35] Balouiri M., Sadiki M., Ibnsouda S.K. (2016). Methods for in vitro evaluating antimicrobial activity: A review. J. Pharm. Anal..

[36] National Committee for Clinical Laboratory Standards (2000). Methods for Dilution Antimicrobial Susceptibility Tests for Bacteria That Grow Aerobically.

